# Mediterranean diet and cognitive health: Initial results from the Hellenic Longitudinal Investigation of Ageing and Diet

**DOI:** 10.1371/journal.pone.0182048

**Published:** 2017-08-01

**Authors:** Costas A. Anastasiou, Mary Yannakoulia, Mary H. Kosmidis, Efthimios Dardiotis, Giorgos M. Hadjigeorgiou, Paraskevi Sakka, Xanthi Arampatzi, Anastasia Bougea, Ioannis Labropoulos, Nikolaos Scarmeas

**Affiliations:** 1 Department of Nutrition and Dietetics, Harokopio University, Athens, Greece; 2 Eginition Hospital, 1st Neurology Clinic, Department of Social Medicine,Psychiatry and Neurology, National and Kapodistrian University of Athens, Athens, Greece; 3 Laboratoty of Cognitive Neuroscience, School of Psychology, Aristotle University of Thessaloniki, Thessaloniki, Greece; 4 School of Medicine, University of Thessaly, Larissa, Greece; 5 Athens Association of Alzheimer’s Disease and Related Disorders, Marousi, Greece; 6 Eginition Hospital, 1st Neurology Clinic, Athens, Greece; 7 Biomedicine Diagnostic Laboratory, Athens, Greece; 8 Taub Institute for Research in Alzheimer’s Disease and the Aging Brain, the Gertrude H. Sergievsky Center, Department of Neurology, Columbia University, New York, New York, United States of America; INSERM, U897, FRANCE

## Abstract

**Background:**

The Mediterranean dietary pattern has been associated with a decreased risk of many degenerative diseases and cognitive function in particular; however, relevant information from Mediterranean regions, where the prototype Mediterranean diet is typically adhered to, have been very limited. Additionally, predefined Mediterranean diet (MeDi) scores with use of a priori cut-offs have been used very rarely, limiting comparisons between different populations and thus external validity of the associations. Finally, associations between individual components of MeDi (i.e., food groups, macronutrients) and particular aspects of cognitive performance have rarely been explored. We evaluated the association of adherence to an a priori defined Mediterranean dietary pattern and its components with dementia and specific aspects of cognitive function in a representative population cohort in Greece.

**Methods:**

Participants from the Hellenic Longitudinal Investigation of Ageing and Diet (HELIAD), an on-going population-based study, exploring potential associations between diet and cognitive performance in a representative sample from Greek regions, were included in this analysis. Diagnosis of dementia was made by a full clinical and neuropsychological evaluation, while cognitive performance was assessed according to five cognitive domains (memory, language, attention-speed, executive functioning, visuospatial perception) and a composite cognitive score. Adherence to MeDi was evaluated by an *a priori* score (range 0–55), derived from a detailed food frequency questionnaire.

**Results:**

Among 1,865 individuals (mean age 73±6 years, 41% male), 90 were diagnosed with dementia and 223 with mild cognitive impairment. Each unit increase in the Mediterranean dietary score (MedDietScore) was associated with a 10% decrease in the odds for dementia. Adherence to the MeDi was also associated with better performance in memory, language, visuospatial perception and the composite cognitive score; the associations were strongest for memory. Fish consumption was negatively associated with dementia and cognitive performance positively associated with non-refined cereal consumption.

**Conclusions:**

Our results suggest that adherence to the MeDi is associated with better cognitive performance and lower dementia rates in Greek elders. Thus, the MeDi in its a priori constructed prototype form may have cognitive benefits in traditional Mediterranean populations.

## Introduction

Population ageing is accelerating worldwide, mainly due to the increase in life expectancy, but also the decrease in birth rates [1]. Although this can be seen as a successful result of the improvements in health care as well as in public health policies, it also creates new challenges to society and health systems, since it increases the rates of age-related degenerative conditions. The progressive decline in cognitive function in the elderly is a major manifestation of the ageing progress. When accompanied by loss of independence it affects the quality of life of the individual but also has various societal and economic costs. Furthermore, dementia has emerged as a major non-communicable disease in the elderly. In 2015, dementia affected more that 47 million people worldwide and the number is expected to triple by 2050 [2]. Beyond the direct cost of dementia itself, a two and a half fold increase in overall mortality risk has been estimated for people with dementia [3]. It should be noted, however, that studies that have compared cohorts in the same population have found that later born populations have lower risk of prevalent dementia, providing some indication that prevalence may be declining [4–7].

The identification of factors that may slow down the decline in cognitive function or even prevent dementia is crucial in terms of public health policy. Nutrition is a modifiable factor that has been consistently associated with cognition at various levels, including intake of specific nutrients or food ingredients [8], consumption of specific foods [9] or specific dietary patterns [10]. Due to the complex biological interactions between different components of a diet, it has been proposed that the use of a whole-diet approach and the study of specific dietary patterns may provide advantages in understanding the role of diet in chronic diseases, including cognitive impairment in the elderly [11].

The MeDi pattern has been associated with a protective effect for many chronic diseases. In the area of brain health, despite some negative studies [12–19], a positive relationship between MeDi adherence and reduced risk for cognitive decline or dementia has been demonstrated by most cross-sectional and prospective epidemiological studies [20–28] and some meta-analyses [29–31]. Additionally, two clinical trials (both part of PREDIMED) with an extended period of follow up support the association between MeDi and cognitive performance [32, 33], while a short duration intervention (i.e. 6 months) in a relatively small number of subjects failed to find an association [34].

One of the methodological issues in the current literature includes the evaluation of adherence to the MeDi, which may limit the generalizability of the results [35]. Specifically, some of the scores that have been used so far are population-specific and cannot be directly compared with scores computed in different samples. The use of specific cut-off points in the context of a score addresses this limitation, measuring adherence to a pre-defined Mediterranean dietary pattern [35]. Additionally, associations between individual components of the MeDi (i.e., specific food groups, macronutrients) and cognitive function have not been adequately explored.

Regarding the influence of diet patterns on cognition, many of the previous studies have focused on dementia as the major clinical outcome, and, to a lesser extent, on detailed cognitive functioning. Furthermore, some of the previous studies have either evaluated cognitive function with a relatively limited cognitive assessment battery (instead of an extensive neuropsychological evaluation) [14, 16, 17, 19], that does not allow a comprehensive assessment of cognitive functioning which may alter our ability to detect subtle associations between nutrition and cognition and to explore relations with particular cognitive domains. Additionally many of them have not explored in detail the relationship between the MeDi and particular cognitive domains. Also, analyses in some of the previous studies were not fully adjusted for all necessary potential confounding variables. Finally, associations between diet, particularly adherence to the MeDi pattern, and cognitive performance have received limited attention in Mediterranean populations. The only relevant study conducted in a Greek population was the EPIC-Greece cohort that assessed cognitive function only through the Mini Mental State Examination Test [14].

Our aim in undertaking the present study was to explore comprehensively the relationship between the MeDi pattern and its components, and specific domains of cognitive functioning in a representative Greek population of elderly adults, a population with a life-long Mediterranean overall lifestyle. Adherence to MeDi was defined by an *a priori*,non population-specific index, and cognitive function was assessed by a comprehensive clinical neuropsychological evaluation of each participant, rather than a limited cognitive screening assessment.

## Methods

### Participants

Adults over the age of 64 years from the HELIAD study were included in the present analysis. The study is a large-scale, multidisciplinary investigation involving the evaluation of a substantial number of factors relevant to dementia. It is being conducted in Larissa and Marousi, two cities in Greece and participants were selected through random sampling from municipality records. Before entering the study, volunteers provided written informed consent. The study protocol was approved by the University of Thessaly and the National and Kapodistrian University of Athens Ethics Committees. Qualified neurologists, trained neuropsychologists and dieticians administered all questionnaires and conducted face-to-face interviews. Depending on participants’ wishes, these sessions took place at day care centers for the elderly, the participants’ homes, etc. Overall, the study ascertained exhaustive information pertaining to domains including demographics, medical history, neurological, psychiatric, and neuropsychological assessment, anthropometry, social life, nutrition, sleep and lifestyle patterns. The full examination had a total duration of approximately two hours per participant. The caregivers assisted, in case the interviewed individual was unable to provide all the information requested. Details on the scope of the study, population, design, recruitment procedures and participation rates have been presented elsewhere [36]. Below we provide some additional details on selected aspects of the evaluation that pertain to the particular analyses of this manuscript.

### Clinical evaluation

Participants provided information regarding all previous neurological conditions, medical problems and illnesses, current medications, hospitalizations, surgeries and injuries, including traumatic brain injury. We also collected information regarding the medical history of the participants’ first-degree relatives, with particular attention to neurological diseases. Changes in performance of daily activities and self-care habits that require physical capacity and cognitive functioning, in particular memory, comprehension, calculations and visuospatial orientation, were measured using the Blessed Dementia Scale [37]. The participants’ ability to use the telephone and transportation, medication management and handling of finances independently was assessed using the Lawton Instrumental Activities of Daily Living scale (IADL) [38].

A structured memory complaint questionnaire was used to assess subjective memory problems and forgetfulness. Participants reporting memory impairment were further interviewed about the onset, duration, course and extent of their symptoms. In particular, they were asked about the approximate time their memory problems started and if someone else has also noticed their difficulties with memory. In addition, they provided information regarding the nature of symptom onset (abrupt versus gradual, insidious onset), the clinical course over time (stable, continuous decline, stepwise deterioration, fluctuating cognition) and the extent of memory difficulties (remembering things they have just heard, names of relatives, word finding difficulties, outings, shopping, handling money). Finally they were asked whether the onset of their memory complaints coincided with the occurrence of any medical, emotional or physical event or with a recent stroke, tremor or gait problems.

Participants were screened for neuropsychiatric symptoms using the 15-item version of the Geriatric Depression Scale (GDS) [39, 40], the 7-item anxiety subscale of the hospital Hospital Anxiety and Depression Scale [41–43], selected items from the Columbia University Scale for Psychopathology in Alzheimer's Disease (CUSPAD) [44] and the Neuropsychiatric Inventory (NPI) [45].

A standardized physical examination was conducted for each participant. Neurologists carried out a structured neurological examination and neurological findings were documented. The presence and severity of parkinsonian signs and symptoms were assessed with the motor examination section of the Unified Parkinson’s Disease Rating Scale [46]. Participants were interviewed for past diagnosis of stroke or transient ischemic attack (TIA) or a history of neurological symptoms consistent with stroke or TIA. When there was a history of stoke/TIA or symptoms suggestive of cerebral ischemia, further information regarding the date of onset, duration, constellation of symptoms, admission to hospital or any kind of rehabilitation treatment was elicited [47]. Medical records, when available, were also reviewed to classify stoke subtypes according to the TOAST criteria [48]. In addition, participants reporting memory deterioration were also asked if they had a sudden loss of cognitive function around the time they had a stroke and if they noticed a fluctuating or stepwise progression of their symptoms. Based on information both from the medical history and the neurological examination, we calculated the Hachinski Ischemia Scale score, which is a useful screening tool for vascular dementia [49, 50]. To identify clinical features characteristic of Dementia with Lewy Bodies (DLB) we used the Semi-quantified Clinical Fluctuating Cognition Rating Scale, which evaluates fluctuations in the level of consciousness and/or cognition [51]. In addition, a structured questionnaire was designed to determine presence of core (fluctuation of alertness/cognition, visual hallucinations, parkinsonism), suggestive (REM sleep behavior disorder, severe neuroleptic sensitivity) or supportive features (repeated falls and syncope, transient alteration of consciousness, severe autonomic dysfunction, hallucinations in other modalities, systematised delusions, depression) of the revised DLB diagnostic criteria [52].

### Neuropsychological evaluation and cognitive scores

Participants received a comprehensive neuropsychological assessment including all major cognitive domains. Specifically, we examined the following cognitive domains: Orientation (Mini Mental State Exam) [53], Verbal and Non-verbal Memory [Greek Verbal Learning Test [54] [54], GVLT; including five learning trials of a 16-item shopping list of semantically related items; this measure has strong internal reliability (Cronbach’s α = .84) and good diagnostic utility (area under the ROC curve estimates at approximately 0.7 on three variables), Medical College of Georgia Complex Figure Test (MCG)–immediate and delayed recall of an abstract line drawing] [55], Language [semantic and phonological verbal fluency (categories: objects and the letter A; this measure has good diagnostic validity in a Greek sample of healthy adults and psychiatric and neurological patients) [56]; subtests of the Greek version of the Boston Diagnostic Aphasia Examination short form, namely, the Boston Naming Test-short form, and selected items from the Complex Ideational Material Subtest, to assess verbal comprehension and repetition of words and phrases (these measures have good discriminant validity) [57]], Visuoperceptual Ability [Benton’s Judgment of Line Orientation [58, 59] abbreviated form, we used every third item); MCG Complex Figure Test copy condition, Clock Drawing Test [60] (this measure has good diagnostic validity)], Attention and Information Processing Speed [Trail Making Test-Part A (TMT) (this measure had good diagnostic validity in a Greek sample of healthy adults and neurological patients) [61]], Executive Functioning [TMT-Part B; verbal fluency; Anomalous Sentence Repetition (created for the present investigation); Graphical Sequence Test; Motor Programming [62] (the last two based on Luria’s method); months forwards and backwards], and a gross estimate of Intellectual level [a Greek multiple choice vocabulary test, with good construction validity = 0.713 [63]].

The scores of each cognitive test were converted into z-scores using mean and standard deviation values of the non-dementia group. Subsequently, z-scores of individual neuropsychological tests were averaged to produce domain z-scores in memory, language, attention-speed, executive and visual-spatial functioning. Decision on grouping of neuropsychological tests was based on a priori neuropsychological knowledge of particular cognitive functions that each test reflects. Furthermore, domain z-scores were averaged in order to calculate a composite z-score.

### Diagnoses

Diagnoses were reached through diagnostic consensus meetings of all the researchers and main investigators, both neurologists and neuropsychologists, involved in the project. In particular, the diagnosis of dementia and its subtypes was based on DSM-IV-TR criteria [64] and the designation of probable or possible AD was made according to the National Institute of Neurological and Communicative Disorders and Stroke/Alzheimer Disease and Related Disorders Association (NINCDS/ADRDA) criteria [65]. The diagnosis of vascular dementia was based on a history or clinical evidence of stroke, the presence of a clear temporal relation between stroke and the onset of dementia and the Hachinski Ischemia Scale score [49]. Lewy body and frontotemporal dementias were diagnosed based respective criteria [66, 67]. Quantification of the staging of dementia was performed by means of a semi-structured interview of the Clinical Dementia Rating Scale (CDR), which globally assesses six domains of cognitive and functional performance [68].

### Dietary assessment

Dietary intake was evaluated with a semi-quantitative food frequency questionnaire that has been validated for the Greek population [69]. The questionnaire was completed by each participant with the aid of an experienced investigator and when necessary with the aid of the caregiver. Responses were converted to daily intakes of specific food items and were extrapolated into macronutrient intakes. Energy intake was calculated by summing energy intake from macronutrients and alcohol, assuming 4 kcal/g for carbohydrates and proteins, 9 kcal/g for lipids and 7 kcal/g for alcohol. Dietary intake was also grouped into food groups featuring the core foods of the Greek diet [70]. Adherence to the MeDi pattern was evaluated through the Mediterranean Dietary Score (MedDietScore), proposed by Panagiotakos et al.[71]. The scoring is based on the weekly consumption of eleven food groups and an individual score for each component is calculated, ranging from 0–5. For the consumption of items that are presumed to closely characterize the Mediterranean pattern (i.e. non-refined cereals, fruits, vegetables, legumes, potatoes, fish and olive oil), individuals who reported no consumption were assigned a score of 0, and scores of 1–5 are assigned for rare to daily consumption. For the consumption of foods that are presumed to diverge from this diet pattern (i.e. meat and meat products, poultry and full-fat dairy products), participants were assigned scores on a reverse scale (i.e. from 5 when they reported no consumption to 0 when they reported almost daily consumption). For alcohol intake, a score of 5 was assigned for consumption of less than 300 ml of alcohol/d, a score of 0 was assigned for no consumption or for consumption of 700 ml/d and scores of 4–1 were assigned for consumption of 600–700, 500–600, 400–500 and 300–400 mL/d (100 mL have 12 g of ethanol concentration), respectively. The total score ranged from 0 to 55, with higher values indicating greater adherence to the Mediterranean dietary pattern. This score aims to address one of the limitations of the Mediterranean scores, as the frequency of consumption of each food group part of the score is used as cut-off with respect to the traditional MeDi pattern (i.e. frequency of consumption is compared to predefined scores assumed to be close or away from the MeDi pattern and not to the consumption of the specific population) [35]. In the analyses of the present paper, the MedDietScore was used either as a continuous variable, or as quartiles, where the first quartile (i.e. lowest adherence to the Mediterranean Diet) served as the reference group and was compared to the other quartiles, i.e. Q2, Q3 and Q4, with the last one indicating the greatest adherence to the Mediterranean Diet.

### Other assessments

Height and weight were measured on a levelled platform scale and a wall-mounted stadiometer, to the nearest 0.5 kg and 0.5 cm, respectively. Body Mass Index (BMI) was calculated by dividing the weight in kilograms by the height in meters squared. The presence of clinical co-morbidities was recorded, as part of the medical history, through the use of a questionnaire containing 23 clinical conditions. Education was expressed as total years of education. ApoE genotyping, available for 1,247 participants was performed in genomic DNA extracted from blood buffy coat, using Qiamp DNA Blood Midi Kits (Qiagen, Venlo, Netherlands)[72]. ApoE genotyping was expressed as a trichotomous variable (no e4 allele, one e4 allele, two e4 alleles).

### Statistical analysis

Continuous variables are presented as mean values ± 1 standard deviation and categorical variables as relative (%) frequencies. Differences among groups were tested through analysis of variance for continuous variables and Pearson’s x^2^ for categorical variables. The associations between cognitive status (i.e. dementia versus no cognitive impairment; dependent variable) and MedDietScore (independent variable) were evaluated by logistic regression analysis. MedDietScore was entered into the models both as a continuous variable, as well as in categorical form (comparing the first versus other quartiles). Associations between z-scores of cognitive performance and MedDietScore or its components (expressed as servings of the specific food groups per day) were examined with linear regression analyses. In all analyses age, sex, education, number of clinical co-morbidities and energy intake were entered as potential confounders. In a supplementary analysis, ApoE genotype was also used as a potential confounder in the subgroup of the sample that genotyping was available. We also performed supplementary analyses by limiting dementia cases to those with Alzheimer’s disease only.

## Results

Of the 1,864 participants in the current analysis, 90 (5%) were diagnosed with dementia **(Table 1)**. Sixty-eight cases were diagnosed as Alzheimer’s disease related dementias and 22 cases as other dementias. Individuals with dementia were slightly older and reported fewer total years of education than those without dementia. Additionally, individuals with dementia had fewer clinical co-morbidities and reported lower energy intake, compared to those without dementia. No differences were found between groups in macronutrient intake, expressed as percentages of energy intake; participants with dementia consumed, however, less protein (expressed as grams per day). Comparisons of participant characteristics by quartiles of MedDietScore revealed that participants in the fourth quartile of MedDietScore were slightly older, had decreased BMI, were more educated and reported higher energy intake **(Table 2)** when compared to the other quartile groups.

**Table 1:** Demographic, anthropometric, medical characteristics and dietary intake of the study participants by cognitive status.

	Non-dementia (N = 1,774)	Dementia (N = 90)	All (N = 1,864)	p-value
Age, years	72.7±6.0	78.6±5.9	73.0±6.1	**<0.001**
BMI, kg/m^2^	29.0±4.7	28.5±4.8	29.0±4.7	0.378
Waist circumference, cm	100.8±13.4	100.8±11.4	100.8±13.3	0.996
Sex, %male	40.4	45.6	40.6	0.328
Education, years	7.7±4.7	6.5±5.3	7.6±4.8	**0.019**
Number of clinical co-morbidities	2±2	3±2	2±2	**0.020**
Energy Intake, kcal/day	1,896±527	1,742±479	1,889±525	**0.010**
Carbohydrates, % energy	39±6	39±7	39±6	1.000
Protein				
% of energy	14±3	14±3	14±3	0.377
g/day	67±20	61±20	66±20	**0.008**
Lipids, % of energy	47±7	47±7	47±7	0.743

Values are means ± SD or percentages

**Table 2:** Demographic, anthropometric, medical characteristics, dietary intake and cognitive status of the study participants by quartiles of the Mediterranean dietary score.

	1^st^ quartile, score: 19–31 (N = 416)	2^nd^ quartile, score: 31–34 (N = 432)	3^rd^ quartile, score: 34–37 (N = 470)	4^th^ quartile, score: 37–46 (N = 485)	p-value
Age, years	73.9±5.9	73.3±6.0	72.3±6.7^a^	72.5±5.8^a^	**<0.001**
BMI, kg/m^2^	29.4±5.0	29.3±5.0	28.6±4.4	28.7±4.3	**0.014**
Waist circumference, cm	102±14	101±14	100±13	101±13	0.343
Sex, %male	26.0	33.6	46.4	52.8	**<0.001**
Education, years	6.7±4.4	7.1±4.5	8.1±4.9^a^^,^^b^	8.7±4.9^a^	**<0.001**
Number of clinical co-morbidities	2±2	2±2	2±2 ^b^	2±2	**0.012**
Energy Intake, kcal/day	1,708±501	1,869±526^a^	1,932±519^a^	2,022±507^a^^,^^b^^,^^c^	**<0.001**
Carbohydrates, % energy	37±7	38±7	38±6	40±6^a^^,^^b^^,^^c^	**<0.001**
Protein					
% of energy	15±3	14±2 ^a^	14±3 ^a^	14±2^a^^,b^	**<0.001**
g/day	63±20	66±20	67±21 ^a^	69±19 ^a^	**0.001**
Lipids, % of energy	48±7	48±7	48±7	46±6^a^^,^^b^^,^^c^	**<0.001**
Dementia diagnosis, %	32	35	20	14	**0.003**

Values are means ± SD or percentages

^a, b, c^ indicate statistically significant difference compared to value in the 1^st^, 2^nd^ and 3^rd^ quartiles of MedDietScore, respectively

### MeDi and dementia

Participants diagnosed with dementia had slightly lower total MedDietScore, compared to individuals without dementia (31.8±4.5 versus 33.8±4.3 respectively, p<0.001). Prevalence of dementia was lower in individuals categorized in the higher quartile of MedDietScore, compared to other quartiles **(Table 2)**. For each additional unit of MedDietScore we noted 8% less likelihood of being in the dementia group in the fully adjusted model [odds ratio of 0.920 (0.870–0.974)] **(Table 3)**. In the same model, the odds ratio for age was 1.165 (1.118–1.214); thus the protective effect of approximately a two-unit increase in MedDietScore could counterbalance the effect of one year of cognitive aging. Similarly, the odds ratio for dementia was 65% lower for the individuals in the higher quartile of MeDi adherence, compared to those in the lowest one, in the fully adjusted model.

**Table 3:** Results from logistic regression analysis that evaluated the association between the Mediterranean dietary score and dementia.

Model	MedDietScore as a continuous variable		Quartiles of MedDietScore		
	OR (95% CI)	p-value	OR (95% CI)	p-value	p for trend
Unadjusted	0.903 (0.859–0.950)	**<0.001**	Q1: 1 (reference)		**0.001**
			Q2: 1.040 (0.599–1.805)	0.890	
			Q3: 0.529 (0.279–1.000)	0.050	
			Q4: 0.348 (0.170–0.713)	**0.004**	
Adjusted ^1^	0.909 (0.860–0.960)	**0.001**	Q1: 1 (reference)		**0.005**
			Q2: 1.060 (0.594–1.894)	0.843	
			Q3: 0.601 (0.307–1.177)	0.138	
			Q4: 0.384 (0.181–0.816)	**0.013**	
Fully adjusted ^2^	0.920 (0.870–0.974)	**0.004**	Q1: 1 (reference)		**0.019**
			Q2: 1.175 (0.651–2.119)	0.593	
			Q3: 0.672 (0.340–1.329)	0.253	
			Q4: 0.449 (0.208–0.969)	**0.041**	

^1^ age, sex, education and number of clinical co-morbidities were entered as potential confounders

^2^ further adjusted for energy intake

### Supplementary analyses

Since apoE genotype may be a significant factor in the development of dementia, as a sensitivity analysis we repeated these analyses in the subgroup where apoE genotyping was available, by adding apoE genotype as a confounder in fully adjusted models. When MedDietScore was expressed as a continuous variable, the odds for dementia remained significant and similar to the total sample [0.878 (0.812–0.948), p = 0.001]. When MedDietScore was expressed in quartiles, the odds for dementia in the forth quartile, compared to the first quartile, was also statistically significant in the fully adjusted model [0.292 (0.091–0.944), p = 0.040].

Because Alzheimer’s disease is by far the most common type of dementia, we recalculated the models for the odds for Alzheimer’s disease related dementia only, removing participants with other types of dementia from the analyses, and observed an almost identical association [0.914 (0.855–0.977), p = 0.008], as when all dementias were included.

### MeDi and cognitive performance

#### Total sample

With the exception of attention-speed, all other cognitive domains (memory, language, executive functioning, visuοspatial perception) and composite z-score were positively associated with the MedDietScore in adjusted models **(Table 4)**. These associations remained significant even after further adjustment for energy intake, with the exception of visual-spatial domain, where the association was of borderline significance (p = 0.059). By comparing beta coefficients of MedDietScore and age in the fully adjusted model for the composite score (0.010 and -0.041, respectively), it could be argued that every 4-unit increase in the MedDietScore could counterbalance the negative effect of 1 year of cognitive aging.

**Table 4:** Results from multiple linear regression analysis that evaluated the association between the Mediterranean dietary score and z scores of cognitive performance.

Domain/model	All participants		Excluding participants diagnosed with dementia		Excluding participants diagnosed with dementia and MCI^1^	
	beta ± SE	p-value	beta ± SE	p-value	beta ± SE	p-value
Memory						
Unadjusted	0.029±0.005	**<0.001**	0.025±0.005	**<0.001**	0.023±0.005	**<0.001**
Adjusted	0.018±0.005	**<0.001**	0.015±0.004	**0.001**	0.013±0.004	**0.003**
Fully adjusted	0.015±0.005	**0.001**	0.012±0.005	**0.010**	0.012±0.005	**0.011**
Language						
Unadjusted	0.037±0.005	**<0.001**	0.032±0.005	**<0.001**	0.029±0.005	**<0.001**
Adjusted	0.014±0.004	**<0.001**	0.011±0.004	**0.003**	0.009±0.004	**0.012**
Fully adjusted	0.011±0.004	**0.007**	0.007±0.004	0.055	0.006±0.004	0.094
Attention—Speed						
Unadjusted	0.030±0.007	**<0.001**	0.023±0.006	**<0.001**	0.019±0.006	**0.003**
Adjusted	0.002±0.006	0.684	-0.003±0.006	0.588	-0.055±0.005	0.355
Fully adjusted	-0.003±0.006	0.579	-0.009±0.006	0.103	-0.010±0.006	0.066
Executive						
Unadjusted	0.030±0.005	**<0.001**	0.024±0.004	**<0.001**	0.023±0.004	**<0.001**
Adjusted	0.011±0.004	**0.006**	0.005±0.004	0.174	0.005±0.004	0.146
Fully adjusted	0.008±0.004	**0.049**	0.002±0.004	0.532	0.003±0.004	0.410
Visuospatial						
Unadjusted	0.036±0.005	**<0.001**	0.032±0.005	**<0.001**	0.026±0.005	**<0.001**
Adjusted	0.013±0.005	**0.006**	0.012±0.004	**0.006**	0.008±0.004	0.067
Fully adjusted	0.010±0.005	0.059	0.009±0.005	**0.037**	0.006±0.005	0.194
Composite						
Unadjusted	0.035±0.005	**<0.001**	0.028±0.004	**<0.001**	0.025±0.004	**<0.001**
Adjusted	0.014±0.004	**<0.001**	0.009±0.003	**0.007**	0.007±0.003	**0.030**
Fully adjusted	0.010±0.004	**0.007**	0.005±0.003	0.139	0.004±0.003	0.253

Cells contain beta co-efficient ±SE values. Confounders used in adjusted model: age, sex, education, number of clinical co-morbidities. Fully adjusted model was additionally adjusted for energy intake.

^1^ MCI: Mild cognitive impairment

#### Excluding individuals with dementia

In order to exclude the possibility that the aforementioned associations were affected by misreporting of dietary information in cognitively impaired individuals, we repeated the regression analyses by excluding individuals diagnosed with dementia. Z scores for memory, language, visuospatial domains and composite score were significantly associated with total MedDietScore in adjusted models. Following adjustment for energy intake, the association was attenuated for the composite score and was marginally significant for the language domain (p = 0.055), but remained significant for memory and visuospatial domains.

#### Excluding individuals with dementia and MCI

In order to be even more conservative, we repeated the analyses by further excluding individuals with mild cognitive impairment (N = 223). In the adjusted models, significant associations were still observed between MedDietScore and memory, language and total composite z-score. In fully adjusted models (including energy intake), only the relationship between memory z-score and MedDietScore remained significant.

### Cognition and individual components of MeDi

Individuals with dementia had lower fruits, vegetables and fish consumption, as compared to individuals without dementia **(Table 5)**. In fully adjusted models, a significant negative association was observed between cognitive status (dementia or no dementia) and fish consumption [odds ratio of 0.311 (0.147–0.658)]. Thus, for every serving of fish per day, a reduction in the risk of dementia by 68.9% was observed or, alternatively, 1 serving of fish per week was associated with a 9.8% reduction in the risk of dementia. The total composite score was positively associated with non-refined cereal consumption (beta coefficient of 0.059±0.021) in the fully adjusted model with beta coefficient for age of -0.042±003; hence the beneficial effect on cognitive performance of an increase of non-refined cereals by a serving per day could be considered equal, in absolute terms, with the detrimental effect of 1.4 years of cognitive aging.

**Table 5:** Individual components of the Mediterranean dietary pattern by cognitive status and their association with cognitive status and cognitive performance.

	means ± SD^1^		Association with cognitive status^2^		Association with cognitive performance ^3^	
	Non-dementia	Dementia	OR (95% CI)	p-value	beta±SE	p-value
Non-refined cereals	0.8±0.8	0.7±0.7	0.902 (0.643–1.266)	0.551	0.059±0.021	**0.004**
Potatoes	0.2±0.2	0.2±0.2	4.171 (0.964–18.049)	0.056	0.003±0.106	0.980
Fruits	2.1±1.3	1.7±1.3*	0.874 (0.703–1.087)	0.225	0.006±0.013	0.652
Vegetables	2.0±1.0	1.7±1.0*	0.869 (0.662–1.139)	0.308	0.016±0.016	0.319
Legumes	0.5±0.3	0.5±0.3	1.054 (0.424–2.622)	0.910	-0.102±0.054	0.059
Fish	0.6±0.4	0.4±0.3*	0.311 (0.147–0.658)	**0.002**	0.065±0.038	0.090
Olive oil	1.4±0.5	1.4±0.5	1.692 (0.984–2.907)	0.057	-0.028±0.033	0.397
Red meats	0.8±0.5	0.7±0.5	0.969 (0.556–1.688)	0.911	0.027±0.035	0.437
Poultry	0.4±0.3	0.4±0.3	1.202 (0.508–2.847)	0.676	0.030±0.052	0.565
Full fat dairy	1.3±0.9	1.4±0.9	1.247 (0.949–1.639)	0.113	-0.024±0.018	0.193
Alcohol	0.4±0.7	0.3±0.7	0.850 (0.524–1.378)	0.509	0.023±0.026	0.360

^1^ values represents consumption expressed in servings/day, except olive oil (expressed as times/day)

^2^ Results from logistic regression with dependent variable: cognitive status (dementia, no dementia); independent variable: each specific component of MeDi.

^3^ Results from linear regression with dependent variable: composite z-score of cognitive performance; independent variable: each specific component of MeDi.

All models presented are fully adjusted (adjusted for age, sex, education, number of clinical co-morbidities, energy intake).

* Indicates statistically significant difference, compared to non-dementia group, at p<0.05.

## Discussion

The MeDi dietary pattern has been associated with various health benefits and decreased risk of many diseases. In this observational analysis of 1,864 elderly individuals from a Mediterranean region (Greece) we found that adherence to this pattern was positively associated with a decreased likelihood of dementia and better cognitive performance in many domains, especially memory. Furthermore, individual components of the MeDi, specifically fish and whole cereals, were associated with better cognitive performance.

Some studies have failed to establish an association between adherence to the Mediterranean diet and cognition. The absence of association in these studies could be attributed to lack of statistical power, i.e. few new cases of diagnosed cognitive impairment at follow-up [12], specific characteristics of the population examined, e.g. individuals with, or at risk of, cardiovascular disease [17] or limitations in the assessment of adherence to the Mediterranean diet by the use of *a posteriori* scores [12, 14, 16, 17]. At the same time, several cohort studies, as well as meta-analyses, have revealed a protective effect of the MeDi against cognitive decline in the elderly [20–26, 29–31]. Our results are in line with these studies, and, in fact, extend current knowledge by establishing a positive association between adherence to the MeDi pattern and domain-specific cognitive performance, even in individuals without dementia. Up to date, the few clinical trials in the area have reported inconsistent results. Two studies in the Spanish population have provided results in favor of an association between MeDi and cognitive function [32, 33], while a 6-month intervention in the Australian population failed to establish an association [34]. Possible explanations for the lack of an association in this study could be the short duration of the intervention, the relatively limited number of participants, or the way MeDi was defined (i.e. based on the Australian food and nutrient profile, rather than a more traditional MeDi definition) resulting in smaller nutritional intervention differences between the experimental and the control groups.

The mechanisms by which adherence to the MeDi could exert a protective effect are not fully understood. It has been proposed that the beneficial effects on cognitive function could be mediated through positive effects on cardiovascular risk factors [73, 74]. Furthermore cerebrovascular mechanisms may be involved. A recent study has found that MeDi was associated with reduced brain infarcts [75] and a lower white matter hyperintensity volume [76] in elderly individuals. Another way by which the MeDi may confer its favorable effects in cognitive function is through its anti-oxidant capacity [77] and anti-inflammatory properties [78]. Inflammation has been associated with compromised vascular health, as well as neuronal damage in the brain, through amyloid peptides accumulation and subsequent activation of microglia and reactive astrocytes [79]. In favor of this potential protective mechanism of MeDi, non-refined cereals consumption, a food group with high antioxidant potential, was independently associated with cognitive performance.

Alzheimer’s disease is a neurological condition where neuro-degeneration and hippocampal atrophy is most prominent, even in early phases of the disease’s development [80]. Diet quality has been shown to be related to the volume of the hippocampus (the brain structure that is mainly associated with learning and memory) [81]. In elderly humans, MeDi has been associated with reduced brain atrophy [82] and reduced amyloid peptides load in the brain [83]. In our study, the relative risk for all dementias was identical with the relative risk for Alzheimer’s disease related dementia, while memory was the only cognitive domain consistently associated with MedDietScore, in fully adjusted models and in all cognitive status groups. Taken together, these observations and our results may indicate that the protective effect of MeDi may be mediated by beneficial effects on neuro-degeneration.

Interactions of diet and health and the effect of diet on disease development are complex and difficult to establish. We consistently observed a positive association between adherence to the MeDi pattern and cognitive function, however the associations of individual components of this pattern with cognitive function were less strong and most of them were attenuated in fully adjusted models. As also noted in other studies [26], it appears that interactions of the components of the MeDi pattern or their additive effect, and thus the overall pattern, may be more important for health enhancement than individual food groups alone.

Regarding individual components of the MedDietScore employed in this study, fish consumption was the only predictor of dementia. A substantial body of evidence exists, supporting an association between fish consumption and dementia [84]. Additionally, non-refined cereals were the only food group that was a significantly associated with cognitive performance. This finding has also been reported in a large prospective study of elderly women, wherein greater whole grain intake was not associated with cognitive trajectories, but was related to higher average global cognition scores [85]. This association may be mediated through the antioxidant potential of the specific food group or its other properties, e.g. factors that coincide with increased fiber consumption. Alternatively, increased consumption of non-refined (versus refined) cereals may be an indicator of an increased effort to adhere to a prudent diet.

Our study has several limitations. This is a cross-sectional analysis that cannot provide causal relationships, only state hypotheses for future explorations. Thus, reverse causality cannot be excluded. Another limitation is the precision in the assessment of dietary intake by a food frequency questionnaire and the possibility that individuals with cognitive impairment may misreport their food consumption. Precise and easily applicable methods for the dietary assessment of such large populations, however, are scarce. Additionally, we took every precaution to limit food consumption misreporting. Specifically, trained dietitians administered the questionnaires and when precise information could not be obtained, the caregiver of the individual assisted in the data collection. We also performed all sensitivity analyses possible in order to exclude misreporting (i.e. excluding not only individuals without dementia, but even those with mild cognitive impairment). Although we took under consideration many confounders, the influence of other confounders not assessed in this study (i.e. residual confounding) cannot be entirely excluded.

At the same time, our study has many strengths. We administered a comprehensive neuropsychological evaluation that included both a full neuropsychological battery and a diagnosis of cognitive impairment based on consensus of the investigators’ team, with use of standard criteria. A major advantage of the study is the scoring system used for estimating adherence to Medi. We used a score that is based on weighting of the selected food groups based on the frequency of consumption (thresholds were chosen according to an a priori hypothesis) and regardless of the consumption by the sample studied [35].Dietary data were also collected with an instrument validated in the population studied. Furthermore, our sample is population representative and includes individuals from both a rural and an urban area. In addition, we adjusted for many potential confounders and we performed all necessary sensitivity analyses.

In conclusion, our data suggest a positive association between adherence to the MeDipattern and dementia absence, as well as specific domains of cognitive performance, especially memory, in a traditional Mediterranean population. It seems that the total dietary pattern, rather than individual components, confers the most beneficial effects. This may be of clinical relevance in the setting of population-based prevention efforts as well as in clinical practice. The completion of follow-up evaluations in HELIAD, studies with more robust design, and especially clinical trials, should confirm these associations and elucidate the mechanisms by which MeDi or its components exert their potential health enhancing effects on the nervous system.
